# Regulation of Oxygen in the Tumor Microenvironment Synergizes with Immunotherapy to Suppress Tumor Progression

**DOI:** 10.3390/jfb15120357

**Published:** 2024-11-25

**Authors:** Shoucheng Wang, Yongjie Chi, Danyang Wang, Kai Zhao, Lianyan Wang

**Affiliations:** 1 Engineering Research Center of Agricultural Microbiology Technology, Ministry of Education, Heilongjiang University, Harbin 150500, China; wangshoucheng3@gmail.com; 2 Key Laboratory of Microbiology, College of Heilongjiang Province, School of Life Sciences, Heilongjiang University, Harbin 150080, China; 3 Taizhou Key Laboratory of Biomedicine and Advanced Dosage Forms, School of Life Sciences, Taizhou University, Taizhou 318000, China; 4 Key Laboratory of Green Process and Engineering, Institute of Process Engineering, Chinese Academy of Sciences, Beijing 100190, China; chiyongjie21@ipe.ac.cn (Y.C.); wangdanyang24@ipe.ac.cn (D.W.); 5 State Key Laboratory of Biochemical Engineering, Institute of Process Engineering, Chinese Academy of Sciences, Beijing 100190, China; 6 School of Chemical Engineering, University of Chinese Academy of Sciences, Beijing 100049, China

**Keywords:** tumor microenvironment, hypoxia, immunosuppression

## Abstract

Hypoxia represents a crucial characteristic of the tumor microenvironment, which is closely related to cell proliferation, angiogenesis, and metabolic responses. These factors will further promote tumor progression, increase tumor invasion, and enhance tumor metastasis potential. A hypoxic microenvironment will also inhibit the activity of infiltrated immune cells in the tumor microenvironment, leading to the failure of cancer immunotherapy. Additionally, the hypoxic tumor microenvironment contributes to resistance to conventional therapies and leads to unfavorable prognoses. This review discusses advancements in strategies aimed at ameliorating tumor hypoxia within the microenvironment and modulating immune cell responses against tumors.

## 1. Introduction

**Figure 1:**
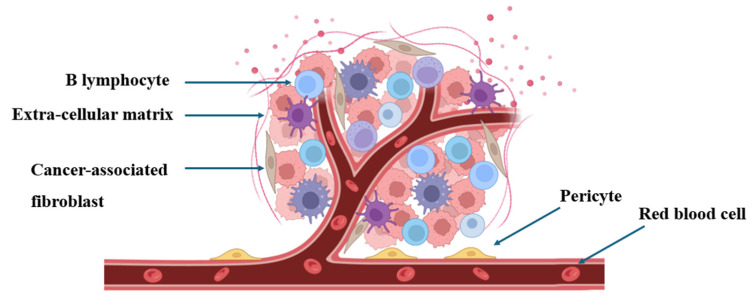
The interaction between immune cells and tumor cells within the TME.

Malignant tumors, commonly referred to as cancer, constitute a disease characterized by the accumulation of somatic cell mutations enabling immortal replication, invasive metastasis, and immune evasion. Solid malignant tumors comprise transformed cells embedded within abundant connective tissue or stroma, thereby forming the tumor microenvironment (TME) [1] (Figure 1). It indicates that the rapid proliferation of tumor cells intensifies oxygen consumption within the TME, ultimately leading to a hypoxic microenvironment. Hypoxia represents a significant feature of the TME, promoting angiogenesis and tumor growth through the upregulation of vascular endothelial growth factor (VEGF) [2], thereby enabling tumor cells to adapt to hypoxic and nutrient-restricted conditions. Moreover, hypoxia alters tumor metabolic responses. Collectively, these factors augment tumor invasiveness, metastasis, and contribute to unfavorable prognoses [3].

Hypoxia influences metabolic alterations in tumor cells, including low pH, high levels of reactive oxygen species (ROS), abnormal vasculature, and proliferative fibrous tissue. These changes favor tumor cell survival, confer anti-apoptotic advantages, inhibit drug penetration, and promote cancer development and drug resistance. Many anti-tumor drugs exert oxygen-dependent cytotoxic effects, primarily through intracellular oxidation to generate free radicals and reactive ROS for tumor cell destruction. Hypoxia induces the expression of relevant target genes, activates multidrug resistance genes, conferring tumor cells with resistance to conventional drug therapies, and rendering them more resistant to tumor treatment drugs. Some conventional cancer treatment methods, particularly those reliant on oxygen, such as chemotherapy, photodynamic therapy, sonodynamic therapy, and radiotherapy, may result in a poor prognosis due to the hypoxic microenvironment. Consequently, oxygen supplementation targeted at tumor sites represents the most effective ways to ameliorate tumor hypoxia and enhance tumor therapy [4].

**Figure 2:**
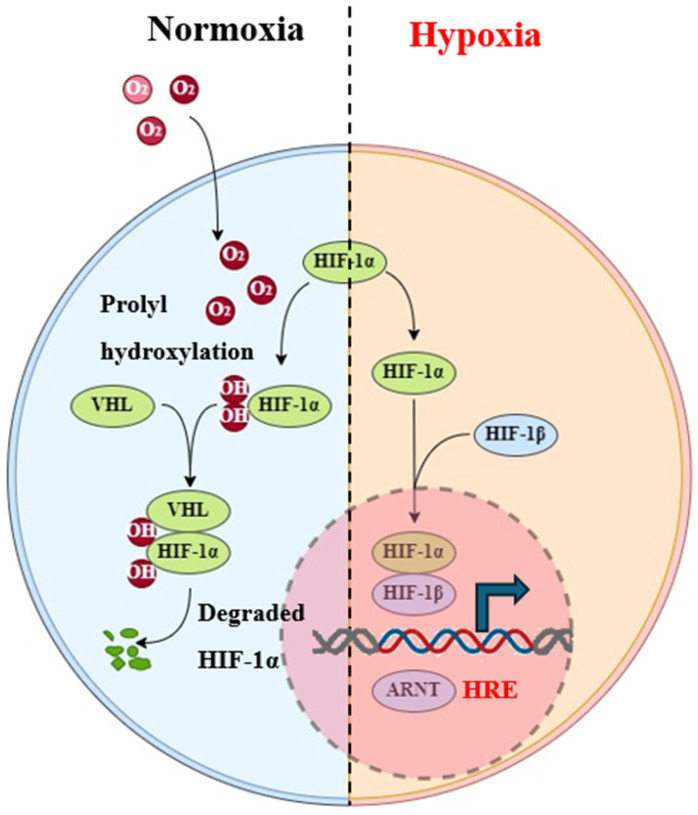
Metabolic processes regulated by HIF-1α in hypoxia and normoxic cells.

The TME undergoes various stages of immune responses, transitioning from immune surveillance to immune escape. Increasing evidence suggests that the hypoxic TME can enhance tumor cell resistance to immune attacks, enabling immune evasion and leading to acquired resistance to immunotherapy. Literature reports indicate that hypoxic regions within tumors are infiltrated by high levels of myeloid-derived suppressor cells (MDSCs), tumor-associated macrophages (TAMs), and regulatory T cells (Tregs) [5,6]. The hypoxic environment suppresses the activation of these infiltrating lymphocytes, resulting in immune suppression and evasion of immune surveillance. Research has shown that a decrease in oxygen levels within the TME activates hypoxia-inducible factors (HIFs) [7], with hypoxia-inducible factor-1α (HIF-1α) participating in hypoxia-related responses within the TME, accelerating malignant tumor progression and playing an important role in tumor pathogenesis [8] (Figure 2). Furthermore, the hypoxic microenvironment can attract lymphoid immune cells, but activated HIF-1α may reduce and deactivate cytotoxic lymphocytes, including NK cells and CD8^+^ T lymphocytes, thereby inhibiting their anti-tumor efficacy [9].

Therefore, by improving hypoxia within the TME, it is possible to alleviate a series of adverse factors associated with the hypoxic microenvironment and potentially facilitate the reversal of the tumor immune microenvironment in subsequent stages.

## 2. Advanced Strategies for Normalizing Tumor Oxygen Levels

Hypoxia is a critical characteristic of the solid malignant TME, which further promotes cancer progression. Therefore, improving the hypoxic state in the TME can reduce the suppressive effect of the tumor on the immune system, which is conducive to improving the effect of immunotherapy.

**Figure 3:**
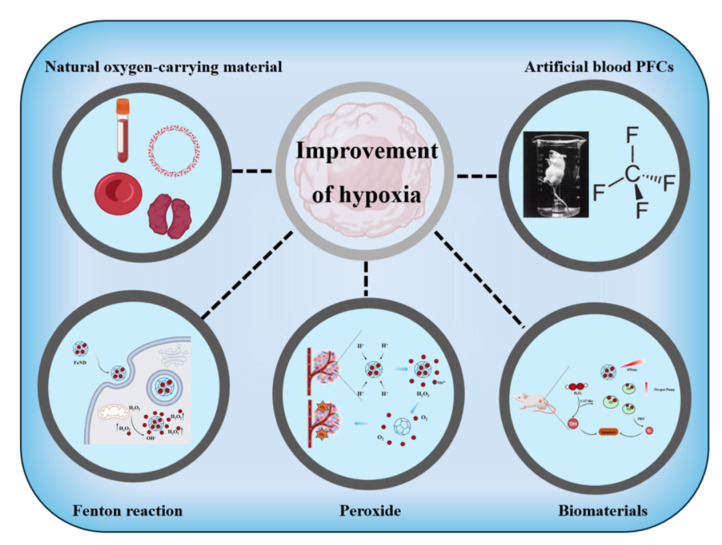
The TME improves the hypoxic pathway.

In recent years, to ameliorate hypoxia within tumors, researchers have developed various materials [10,11], including: (i) natural oxygen-carrying materials for delivery oxygen to tumor sites, such as hemoglobin; (ii) the use of artificial blood substitutes such as perfluorocarbons (PFCs) and their derivatives to carry oxygen to improve hypoxia at tumor sites; (iii) the design of corresponding carrier materials via catalase-like or Fenton-like reactions which produces hydroxyl radicals and oxides with strong oxidation capacity to alleviate hypoxia in the tumor site; (iv) the delivery of inorganic peroxides (such as calcium peroxide) to tumor sites to supplement oxygen levels; and (v) novel biomaterials, such as nanoenzymes, particles, and hydrogels, which can enhance tumor hypoxia alleviation when combined with other tumor treatment modalities, including photodynamic therapy or photothermal therapy (Figure 3).

### 2.1. Natural Oxygen Carriers

Hemoglobin (Hb) is a functional protein present in red blood cells, known for its oxygen transport capabilities. In recent years, researchers have developed a series of chemically conjugated or physically encapsulated Hb-based oxygen carriers for tumor therapy (summarized in Table 1). Yang et al. [12] simultaneously encapsulated Hb and the anticancer drug doxorubicin (DOX) into liposomes (DHL). Compared to DOX-loaded liposomes without Hb, DHL, benefiting from oxygen supplementation, exhibited significantly enhanced cytotoxic activity against cancer cells and tumor growth inhibition. Furthermore, the combination of hemoglobin with tumor therapeutic drugs has been investigated. Le et al. [13] constructed a biomimetic nano-system by combining hemoglobin extracted from red blood cells with the chemical drug sorafenib. Injected into tumor sites under phototherapy, the system not only enhanced oxygenation but also led to a temperature increase of up to 14.0 °C at the tumor site, significantly enhancing the tumor toxicity of chemophototherapy. Han et al. [14] developed a novel Hb-based chemotherapy-photodynamic therapy (PDT) nano-cluster. Compared to unmodified Hb, the nano-cluster mediated chemotherapy-PDT which resulted in a 96.6% reduction in tumor volume, displaying significantly reduced levels of hypoxia markers and anticancer effects at the tumor site.

The presence of extensive hypoxic regions within tumor sites severely impedes drug therapy, and adequate oxygenation is imperative for treating tumors. Inspired by the high oxygen-carrying capacity of hemoglobin and the light-driven activity of erythrocytes, Han et al. [15] proposed a uniform-sized hemoglobin-based microgel (uGel) system resembling red blood cells. This system can release oxygen under severe hypoxic conditions upon near-infrared laser stimulation, assisting UCNPs/Ce6 in generating sufficient reactive ROS, thereby achieving effective photodynamic activity.

In summary, drug development based on Hb carriers holds great promise for alleviating the tumor hypoxia issue.

### 2.2. Synthetic Oxygen-Carrying Organic Compounds

Perfluorocarbons (PFCs) and their derivatives, due to their high biocompatibility, oxygen-carrying capacity, and high hydrophobicity [16], are currently employed as substitutes for artificial blood. Nanostructures containing oxygen based on PFCs can safely transport oxygen to tumor sites and efficiently release it. Additionally, due to the primarily van der Waals forces between PFC and oxygen molecules, PFCs are able to dissolve large amounts of oxygen and be widely utilized in therapeutic strategies for tumor inhibition by improving hypoxia (summarized in Table 1).

Typically, PFC is often emulsified with biomacromolecules to form nanoparticles for oxygen enhancement in synergistic tumor therapy. Song et al. [17] prepared PFC nanoparticles with human serum albumin to form PFC nanoemulsions. When applied to tumors using clinically applicable low-power ultrasound transducers, the oxygenation of tumor tissue increased by approximately 45% compared to non-PFC nanoemulsions, resulting in a fivefold reduction in tumor volume and enhanced photodynamic therapy efficacy. Furthermore, oxygen-carrying PFC can be utilized to construct artificial red blood cells for oxygen transport. Gao et al. [18] studied the use of red blood cell membrane-coated perfluorocarbons as nano-scale artificial red blood cells. Quantitative analysis revealed that the total oxygenation level at the tumor site increased from 1.6% to 24% after 24 h of the injection, significantly improving the oxygenation status at the tumor site.

Sonodynamic therapy utilizes ultrasound irradiation to generate reactive ROS, thereby achieving tumor destruction. However, the severe hypoxic microenvironment of tumors significantly hinders treatment efficacy. Therefore, to enhance effectiveness, Yang et al. [19] prepared a class of covalent organic polymers (COPs). Semi-quantitative analysis revealed that the positive hypoxic area of tumor slices in the COPs group was only 6.2%, significantly lower than the 20.4% in the blank control group. COPs effectively alleviated tumor hypoxia, and combined with low-frequency ultrasound irradiation, they effectively suppressed tumor growth and prevented tumor recurrence.

In summary, there is increasing attention and research focused on using perfluorocarbons for oxygen delivery to improve the hypoxic TME.

**Table 1:** Summary of the studies on oxygen delivery nanomaterials for alleviating hypoxia.

Key Materials	Nanoplatforms	Oxygen Carring Capacity	Mode of Cancer Therapies	Tumor Model	Tumor Oxygenation or Therapeutic Effectiveness	Refs
Hb	DOX-Hb-DHL	4.5 mg/L	chemotherapy	4T1	No hypoxia signals were observed; significant down-regulation of HIF-1α.	[12]
Hb	HUSI NPs	7 g/L	Chemotherapy phototherapy	HCC	Enhance the phototherapy and downregulate expression of HIF-1α.	[13]
Hb	Dox@HPBC	3.75 mg/L	phototherapy PDT	Hela	All groups showed highly reduced HIF-1α expression (<60% compared to that of the no-treatment group).	[14]
Hb	Hb uGels	27 mg/L	PDT	4T1	Effectively elevated the tumor oxygen level (>60%) at 0–30 min.	[15]
PFC	PFC@HSA	1.45 mg/mL	PDT	4T1	The positive hypoxia area remarkably decreased from ∼88% for control group to ∼74% and ∼20%.	[17]
PFC	PFC@PLGA-RBCM	14.43 mg/L	RT	4T1	Severe damage and a high level of tumor cell apoptosis could be observed in the tumor.	[18]
PFC	PFCE@THPP*_PF_*-COPs	6.5 mg/L	SDT	CT26	The positive hypoxic area of tumor sections was only 6.2%, which was significantly lower than that of the blank control group (20.4%).	[19]
PFOB	PFOB@LIP-IR-780	12 mg/L	PTT/PDT	4T1	The oxyhemoglobin signal intensity within the tumor region increases gradually, reaching a maximum 24 h after injection.	[20]
PFP	BPD-PFP	-	PDT	FaDu	The pimonidazole signal is very low, indicating a significant reduction in hypoxia.	[21]
PFC	PFC-PLGA-IR780	16 mg/L	PDT/PTT	4T1	Tumor oxygenation: the green fluorescence of HIF-1α almost disappeared after treat with PFC-PLGA-IR780 combined PDT and PTT.	[22]

### 2.3. Intracellular Autoregulation of Chemical Reactions Improves the Hypoxic State of Tumors

The dysfunctional mitochondria in tumor cells lead to an imbalance between the generation and removal of reactive ROS, including H_2_O_2_. This oxidative stress results in the accumulation of large amounts of H_2_O_2_ at tumor sites. The Fenton reaction [23] exploits the accumulated H_2_O_2_ characteristic of tumor sites. Through the interaction between hydrogen peroxide (H_2_O_2_) and ferrous ions (Fe^2+^), highly reactive hydroxyl radicals (·OH) are generated. The OH radicals produced possess strong oxidizing properties, causing oxidative damage and death to DNA and other biological molecules, thereby achieving the goal of killing tumor cells. This process serves to achieve dual functions of improving tumor hypoxia and inducing tumor cell damage (summarized in Table 2). Selvam et al. [24] proposed a photodynamic therapy (PCT) strategy for cancer treatment utilizing a Fenton reaction induced by near-infrared (NIR) irradiation of Fe-doped nanodiamonds (FeND). The iron oxide within FeND exists in the form of Fe^2+^/Fe^3+^. Under NIR laser irradiation, H_2_O_2_ present at tumor sites can generate more hydroxyl radicals, thereby enhancing the efficacy of PCT. Zhang et al. [25] developed a dual-generation Fenton reagent capable of producing O_2_/OH using CaO_2_/Fe(OH)_3_ nanocomposites. This reagent was co-loaded with Gox molecules into biocompatible liposomes to prepare a nano-composite drug. Under the acidic TME, CaO_2_/Fe(OH)_3_ nanocomposites decompose, producing H_2_O_2_ and Fe^3+^, which subsequently undergo a Fenton reaction. The oxygen generated simultaneously enhances the catalytic efficiency of glucose oxidase (GOx), thereby enhancing the efficacy of starvation therapy (ST) in further eradicating tumors.

The utilization of other metal ions or non-metal catalysts to achieve similar redox processes is referred to as Fenton-like reactions, including transition metals such as manganese, copper, cobalt, and non-metals such as glucose oxidase. Bao et al. [26] developed a metal-organic framework (MOF) integrated with platinum nanoparticles (Pt NPs) and glucose oxidase (GOx) using PCN-224 nanoparticles disguised with red blood cell membranes. When the nano-carrier is engulfed by tumor cells, the surface Pt NPs utilize endogenous H_2_O_2_ to generate oxygen, resulting in 60% remission of hypoxia at the tumor site compared to the control group and enhancing sonodynamic therapy. Radiation therapy, as the most effective means of treating cancer, also experiences reduced efficacy due to tumor hypoxia. Therefore, to enhance the efficacy of radiation therapy, Huang et al. [27] constructed a drug release platform activated by red blood cells and glucose, capable of maintaining a normoxic microenvironment at tumor sites for an extended period and improving the efficacy of repeated radiation therapy.

### 2.4. Inorganic Peroxides

Inorganic peroxides can react with reactive ROS to generate oxygen or scavenge oxygen radicals. Furthermore, they can produce oxygen under other catalytic conditions to ameliorate hypoxia in the TME (summarized in Table 2).

Due to the high reactivity and specificity of manganese dioxide (MnO_2_) nanoparticles towards hydrogen peroxide (H_2_O_2_), as well as their ability to continuously produce oxygen and regulate pH, MnO_2_ nanoparticles have been widely used in TME modulation in recent years. Wu et al. [28] had engineered multifunctional and colloidally stable bioinorganic nanoparticles composed of polyelectrolyte albumin complex and MnO_2_ nanoparticles (A-MnO_2_ NPs). In vitro studies have shown that utilizing the reaction between MnO_2_ and hydrogen peroxide to modulate the TME increased tumor pH from 6.7 to pH 7.2 while also increasing tumor oxygenation by 45%. Intratumoral treatment showed that A-MnO_2_ NPs reacted with H_2_O_2_ at the tumor site to produce oxygen to promote oxygenation of tumor tissues, resulting in more tumor cells dying during radiation and delaying tumor growth, demonstrating enormous potential. Additionally, Liu et al. [29] coated MnO_2_ with ultrathin FeOOH; in TME regulation, MnO_2_ exhibited catalase-like properties, and the oxygen generation assistance catalysis of the FeOOH coating decomposed H_2_O_2_ into oxygen endogenously, alleviating tumor hypoxia and achieving tumor cell killing. Based on these experimental examples, utilizing MnO_2_ nanomaterials to catalyze H_2_O_2_ and alleviate hypoxia, thereby enhancing tumor treatment, has become a promising approach in the TME.

Calcium peroxide (CaO_2_) is rapidly decomposed in the TME to produce H_2_O_2_, and catalase breaks down H_2_O_2_ into oxygen and water. Currently, it is primarily employed in synergistic therapy for hypoxic tumor sites. Liu et al. [30] developed an oxygen self-sufficient liposomal nano-system, where methylene blue and calcium peroxide were encapsulated in the aqueous cavity and hydrophobic layer, respectively. At the tumor tissue site, brief light irradiation activated the photosensitizer methylene blue to induce lipid peroxidation, causing its rupture, releasing CaO_2_, which upon contact with water or weak acids, increased oxygen production, modulated the hypoxic state of the TME, and further enhanced the production of singlet oxygen generated by the photosensitizer under subsequent irradiation, thereby improving the photodynamic therapeutic effect on tumors. Liu et al. [31] prepared calcium peroxide nanoparticles (CP) by reacting CaCl_2_ with H_2_O_2_ under alkaline conditions, followed by synthesizing copolymers containing photosensitizers and siloxane using RAFT polymerization, obtaining copolymer surface-modified CP nanoparticles. These nanoparticles can effectively generate oxygen in the TME, alleviate tumor hypoxia, act as an oxygen self-sufficient platform for enhancing photodynamic therapy, and significantly improve treatment outcomes.

### 2.5. Biomaterials for Improving Hypoxia in Combined Therapy of Cancer

Biomaterials are substances that can be employed for encapsulating active molecules or materials while providing support for combined cancer therapy. Various biomaterials, such as nano-enzymes, hydrogels, and particles, can reshape the TME through transdermal, implantable, and injectable delivery systems (summarized in Table 2) [32].

Li et al. [33] developed the Au@Pt-Ce6-HN-1 nanoplatform, which exhibits dual enzymatic activities resembling catalase and peroxidase. In the TME, it decomposes H₂O₂ to generate oxygen. In vivo experiments demonstrated that this nanoplatform significantly inhibited tumor growth. Following the combination of PDT and PTT, the nanozyme’s oxygen production capability showed outstanding anti-tumor efficacy. Feng Lu’s team [34] designed a semiconductor polymer (PCPDTBT) nanomaterial encapsulated in a mesoporous silica matrix, capable of generating H₂O₂ under hypoxic conditions to alleviate tumor hypoxia. In a mouse tumor model, the nanoplatform combined with laser irradiation significantly inhibited tumor growth. In vivo imaging and histological analysis demonstrated that these nanomaterials effectively accumulated in the tumor site, and the combination of chemotherapy and PDT exhibited high anti-tumor activity.

**Figure 4:**
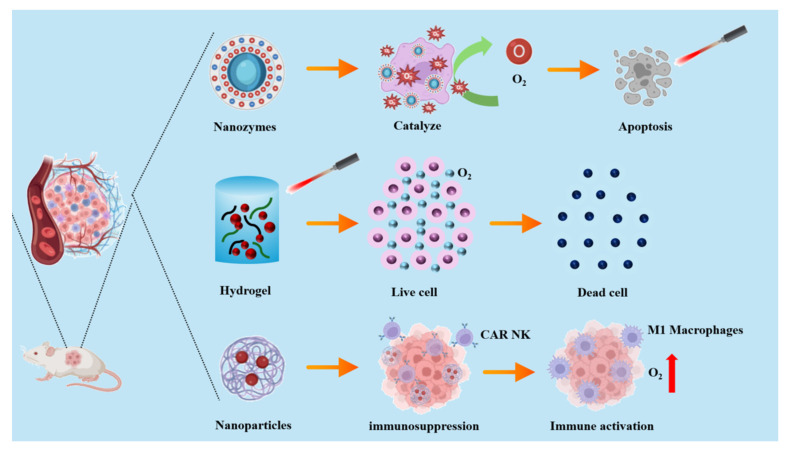
Schematic diagram of the synthesis process and working principle of the agarose@SH/MnO2/Ce6 hydrogel. Effective tumor inhibition was accomplished through enhanced photo-induced tumor therapy on the basis of the relief of tumor hypoxia.

The team led by Wenxue Zhang [35] investigated an in situ injectable nano-composite hydrogel based on chitosan/dextran. This hydrogel effectively inhibits tumor growth through the synergistic effects of drug release and oxygen generation. Furthermore, studies have shown that this hydrogel can modulate macrophage polarization; animal experiments revealed a significant increase in the proportion of M1-polarized macrophages due to the alleviation of hypoxia at the tumor site, ultimately suppressing tumor migration and dissemination. Hou et al. [36] synthesized a gelatin hydrogel containing MnO_2_. Upon near-infrared light irradiation, it promoted the reaction between MnO_2_ and H_2_O_2_, effectively alleviating tumor hypoxia and enhancing the efficacy of photodynamic therapy (PDT)/photothermal therapy (PTT) for tumor treatment (Figure 4).

Jiazhi Duan et al. [37] designed MnO_x_ nanoparticles that can catalyze the excessive production of oxygen from H_2_O_2_ within the TME. Additionally, these nanoparticles achieve targeted delivery by binding to CAR-NK cells via CD56 antibodies. Mouse experiments demonstrated that CAR-NK cells armed with MnO_x_ nanoparticles significantly inhibit tumor growth and extend the survival time of tumor-bearing mice. Furthermore, this study found that this combinatorial therapy increases oxygen levels at the tumor site, enhances immune cell infiltration, and promotes M1 polarization of macrophages, thereby reversing the immunosuppressive TME. David et al. [38] investigated the encapsulation of MnO_2_ nanoparticles within PLGA. When the nanoparticles infiltrate the tumor core, they modulate hypoxia, leading to a reduction in HIF-1α expression. The resulting changes in the microenvironment enhance the functionality of NK cells, thereby eliminating tumor spheroids.

With advancements in nanotechnology, bioengineering, and materials science, the application of biomaterials in tumor hypoxic microenvironments shows significant potential to enhance the efficacy of existing cancer treatments. However, ensuring the biocompatibility, targeting capability, and safety of these biomaterials remains a critical focus for future research.

**Table 2:** Summary of the studies on oxygen generation nanomaterials for alleviating hypoxia.

Key Material	Nanoplatforms	Oxygen Generating Capacity	Mode of Cancer Therapies	Tumor Model	Tumor Oxygenation or Therapeutic Effectiveness	Ref.
Fe	FeND	-	phototherapy	A549	Reducing the cell viability by around 80% in both normoxic and hypoxic conditions.	[24]
Fe	LipoCaO_2_/Fe(OH)_3_-GOx	-	CDT/ST	MDA-MB-231	Downregulate HIF-1α; Kill cancer cells.	[25]
Pt	Pt@PCN-224	33.52 mg/L	SDT	BxPC-3	HIF-1α immunofluorescence intensity is greatly reduced.	[26]
GOx	Endo@GOx-ER	-	RT	4T1	Increased intensity of oxyhemoglobin signal within the tumor region.	[27]
MnO_2_	A-MnO_2_NPs	0.0095 mM	RT	EMT6	45% increase in tumor oxygenation.	[28]
MnO_2_	FeOOH@ MnO_2_	18 mg/L	SDT	MBA-MD-231	Hypoxic signal is diminished in tumor tissue sections.	[29]
CaO_2_	LipoMB/CaO	1.5 mg/L	PDT	4T1	HIF-1α and CA9 expression decreased.	[30]
CaO_2_	NIPAAm-co-VBRB	10 ug/mL	PDT	-	-	[31]
Au Pt	Au@Pt-Ce6-HN-1	25 mg/L	PDT/PTT	TSCC	Elevates oxygen levels at the tumor site.	[33]
PCPDTBT	SP@mSiO_2_-PEG/FeDOX	-	PDT	4T1	Tumor inhibition ratio of ∼85%.	[34]
Hydrogel	CD@CAT	1 mM	-	B16F10	The polarity of TAMs shifts from the M2 phenotype to M1, thereby improving anti-tumor efficacy.	[35]
Hydrogel	agarose@SH/MnO_2_/Ce6	7 mg/L	PDT/PTT	4T1	Tumor suppression rate 93.8%; No HIF-1α fluorescence was observed in tumor sections	[36]
MnO_2_	MnO*_X_*-CD56	-	-	A549-FAP	HIF-1α is greatly reduced in tumor tissue	[37]
MnO_2_	PLGA-MnO_2_NP	-	-	MCF-7	HIF-1α decreased	[38]
Fe/Mn-THPPTK	DOX@Fe/Mn-THPPTK-PEG	12 mg/L	PDT	4T1	Apoptotic cells was eminently increased.	[39]
CaO_2_	CaO_2_@DOX@ZIF-67	3.75 mg/L	CDT	MCF-7	The HIF-1α of group treated with CaO2@DOX@ZIF-67 shown slight green fluorescence.	[40]
Prussian blue	PB@PMO-Ce6	Obvious bubbles	PDT	U87MG	Weak fluorescence signal of the O_2_ probe [Ru(dpp)_3_]Cl_2_ proved the O_2_ production.	[41]

## 3. Normalization of Tumor Oxygen Levels Enhances the Efficacy of Immunotherapy

**Figure 5:**
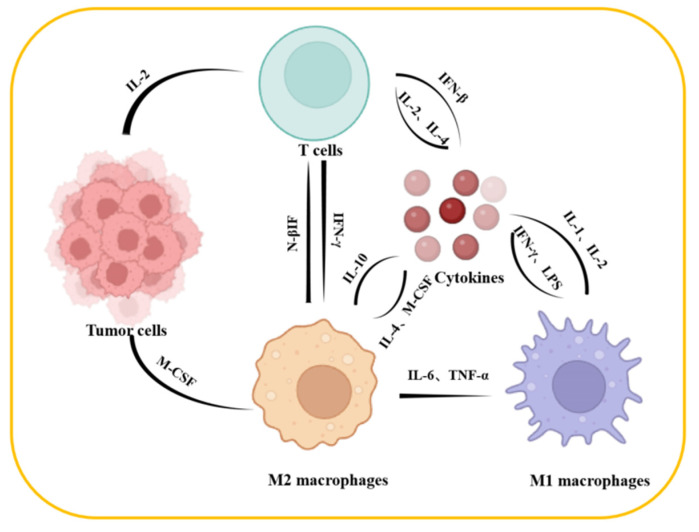
Regulation of tumor immunosuppressive microenvironment.

Modulating the tumor hypoxic microenvironment is the first step to improve tumor immunotherapy, and three regulatory pathways have been reported for the immunosuppressive microenvironment (Figure 5).

One is to enhance the anti-tumor immune response by reducing immunosuppressive factors. For example, Jie Tan’s team [42] designed and constructed calcium phosphate-enhanced iron-based metal organic frameworks (CaP@Fe-MOFs) that regulate adenosine metabolism by promoting adenosine kinase-mediated phosphorylation and alleviating hypoxic TME. In addition, this system can also enhance anti-tumor immune responses through adenosine regulation, including enhancement of T lymphocytes and dendritic cells and reduction in regulatory T cells. Arthur M. The Mercurio [43] reported that the anti-tumor immune response can be enhanced by reducing the targeting of VEGF, and that VEGF promotes the vascular immune barrier through VEGF receptor activity on endothelial cells, which indirectly interferes with immune cell trafficking and leads to immune tolerance at tumor sites, and by targeting VEGF receptors and their drugs, VEGF expression can be effectively reduced and anti-tumor immune responses can be restored. Prof. Hangyu Li et al. [44] systematically reviewed the effects of hypoxia-inducible factor (HIF) on TME-infiltrated cells and the regulation of the TME microenvironment for HIF. Inhibiting and reducing HIF can effectively alleviate the immunosuppressive microenvironment at tumor sites and enhance lymphocyte infiltration. Zhaoting Li [45] developed fluorine-assembled nano-formulations in which perfluorocarbons are highly oxygen-carrying and relieve the hypoxic state of the tumor microenvironment. In addition, the combination of laser irradiation can release chemical prodrugs such as gemcitabine, downregulate glutathione (GSH) content, and also reduce the expression of Foxp3 in Tregs, a transcriptional regulator that is essential for the development and inhibitory function of Tregs, thereby reversing the immunosuppressive function of Tregs. More importantly, downregulating GSH content and upregulating oxygen content have the same effect as photodynamic therapy, which can induce strong immunogenic cell death, thereby promoting dendritic cell maturation and effector T cell activation. In addition, the regulation of HIF-1α and GSH releases Tregs-induced immunosuppressive binding, thereby completely activating the immune anti-tumor response, providing a new immunomodulatory strategy from the perspective of redox regulation.

The second is to achieve an anti-tumor immunity enhancement effect by regulating tumor cell cycle progression or mutant genes. Stanger et al. [46] reported that cell cycle regulation could effectively improve tumor immunosuppression, and that cell cycle progression depended on the coordinated expression and activity of cyclins; cyclin-dependent kinases 4 and 6 (CDK4/6) were particularly promising targets for therapeutic interventions, and the researchers developed CDK4/6 kinase inhibitors which could not only effectively inhibit tumor growth, but also improve the tumor immunosuppressive microenvironment, making it significantly more sensitive to immune checkpoint blockade. Deng [47] found that Lycium barbarum polysaccharide (LBP) could reduce intracellular lipid accumulation by inhibiting the endoplasmic reticulum stress IRE1α-XBP1 pathway, and enhancing the function of tumor-associated dendritic cells (TDCs), thereby stimulating the immune function of T cells and exerting anti-tumor effects. In addition, Laurie H. Glimcher [48] found that the ER stress response factor XPB1 could weaken tumor immunity and promote tumor progression in TDCs. XPB1 was stimulated by lipid peroxidation by-products to activate the biosynthesis program of triglycerides in TDCs, resulting in abnormal lipid accumulation that inhibits the ability of TDCs-presenting antigens to activate T lymphocytes. In addition, Hu et al. [49] constructed a catalytic drug loaded with Fe^2+^-Ru^2+^ mesoporous silica nanoparticles to induce oxidative damage in tumor cells. Oxidatively damaged mtDNA was able to escape from tumor cells and can act as an immunogenic damage-related molecular modality that polarizes TAMs towards the M1 type.

The third is to improve the tumor immunosuppressive microenvironment by reprogramming or regulating the activity of immune cells, including T cells, TAMs, dendritic cells (DCs), etc. For example, the team of Prof. Fu et al. [50] had developed and detected a heterodimer that binds an anti-CTLA-4 antibody targeting regulatory T cells to a CD-47 ligand signal-regulatory protein, which could selectively block CD47 on intratumoral T cells, effectively remove immunosuppressive T cells in the TME, and significantly enhance the anti-tumor immune response. TAMs are highly malleable and can be further polarized into M1 and M2 isoforms, and Shevde et al. [51] had studied the differentiation of M2 to M1 macrophages by changing the metabolic state of the Hedgehog signaling pathway under drug stimulation, thereby promoting a strong immunogenic response to cancer. Gong et al. [52] had designed a programmable release multifunctional hydrogel platform to shape tumor immunogenicity. Atim-3 and Flt-3L would be released at the tumor site in response to ROS; the released Flt-3L would promote the accumulation and proliferation of DCs; and TIM-3 blocked the phenotype of tolerant DC, promoted the infiltration and activation of T lymphocytes, and enhanced the anti-tumor immune response. The experimental results of tumor-bearing mice show that the tumor suppression rate was as high as 90%, and induced systemic anti-tumor immunity.

## 4. Conclusions and Future Perspectives

Malignant tumors remain a significant threat to human health. In order to enhance tumor treatment, it is imperative to first understand the characteristics of the TME. The hypoxic microenvironment is crucial for tumor initiation and progression, posing significant challenges to clinical treatment and prognosis. Consequently, researchers worldwide have conducted in-depth studies on hypoxia improvement, developing numerous methods to ameliorate tumor hypoxia, which, when combined with conventional cancer treatment modalities, have yielded promising results. Furthermore, investigations into tumor-infiltrating lymphocytes within the TME have aimed to disrupt immune tolerance, with some targeting strategies showing notable progress.

It is foreseeable that the integration of hypoxia improvement with immune modulation is anticipated to achieve substantial advances in cancer therapy. We believe that the synergistic effects of direct tumor destruction via hypoxia improvement mechanisms and immune-mediated tumor progression inhibition will lead to remarkable advancements in cancer treatment.

## Data Availability

Not applicable.
